# Beliefs about the Minds of Others Influence How We Process Sensory Information

**DOI:** 10.1371/journal.pone.0094339

**Published:** 2014-04-08

**Authors:** Agnieszka Wykowska, Eva Wiese, Aaron Prosser, Hermann J. Müller

**Affiliations:** 1 Department of Psychology, Ludwig-Maximilians-Universität, Munich, Germany; 2 Institute for Cognitive Systems, Technische Universität München, Munich, Germany; 3 Department of Psychology, George Mason University, Fairfax, Virginia, United States of America; 4 Neuro-Cognitive Psychology Master Program, Ludwig-Maximilians-Universität, Munich, Germany; 5 Centre for Addiction and Mental Health, Research Imaging Centre/PET Centre, Toronto, Canada; 6 Department of Psychological Sciences, Birkbeck College, University of London, London, United Kingdom; Centre de Neuroscience Cognitive, France

## Abstract

Attending where others gaze is one of the most fundamental mechanisms of social cognition. The present study is the first to examine the impact of the *attribution of mind to others* on gaze-guided attentional orienting and its ERP correlates. Using a paradigm in which attention was guided to a location by the gaze of a centrally presented face, we manipulated participants' beliefs about the gazer: gaze behavior was believed to result either from operations of a mind or from a machine. In Experiment 1, beliefs were manipulated by cue identity (human or robot), while in Experiment 2, cue identity (robot) remained identical across conditions and beliefs were manipulated solely via instruction, which was irrelevant to the task. ERP results and behavior showed that participants' attention was guided by gaze only when gaze was believed to be controlled by a human. Specifically, the P1 was more enhanced for validly, relative to invalidly, cued targets only when participants believed the gaze behavior was the result of a mind, rather than of a machine. This shows that sensory gain control can be influenced by higher-order (task-irrelevant) beliefs about the observed scene. We propose a new interdisciplinary model of social attention, which integrates ideas from cognitive and social neuroscience, as well as philosophy in order to provide a framework for understanding a crucial aspect of how humans' beliefs about the observed scene influence sensory processing.

## Introduction

Being inherently embedded in a social environment, humans have developed means to efficiently read out signals that others convey, to optimize social interactions. For example, humans (and other primates [1], [2]) use gaze to communicate intentions, signal behaviorally relevant locations (e.g., of a potential threat), and establish joint attention in social interactions. Since gaze plays such an important social role, the human brain has developed specialized mechanisms enabling detection of gaze direction and attending where others gaze: superior temporal sulcus (STS) encodes gaze direction information (e.g., [3], see also [4] for a review), while gaze-induced attentional orienting is realized through interactions of STS with intraparietal sulcus (IPS, [4]).

In laboratory settings, the mechanism of attending to where others gaze has been examined using *gaze-cueing paradigms* (e.g., [5]–[7]). Typically, gaze cueing involves a centrally presented face, whose eyes shift direction to one of the visual hemifields. Subsequently, a target is presented either at the gazed-at location (validly cued) or at another location (invalidly cued). In line with a common pattern of results in a standard Posner-cueing paradigm (e.g., [8], [9]), target-related performance is typically better for validly, relative to invalidly, cued locations (*cue validity effect*).

A neural mechanism underlying the validity effect has been identified as *sensory gain control* [10], [11], which increases the signal-to-noise ratio for stimuli at attended, relative to other, locations [12], [13]. Sensory gain control has been examined using single-unit neurophysiology [14], neuroimaging [15], and psychophysics [16], providing converging evidence that attention influences sensory processing by amplifying stimulus-related neuronal signals. Based on the event-related potential (ERP) technique of scalp-recorded EEG, the P1-N1 complex at posterior-occipital electrode sites has been identified as the ERP index of the sensory gain control. For example, Mangun and colleagues [10] observed that when spatial attention was deployed to a location, stimuli subsequently presented there elicited enhanced P1 and N1 components relative to stimuli at other locations. The sensory gain mechanism has been studied extensively using a variety of procedures designed to modulate spatial attention: exogeneous cues [17], central cueing [18], [19]; sustained attention [10], or directional gaze [20]. However, the actual sources of attentional control over sensory processing and the question of whether sensory gain is sensitive to task-irrelevant higher-order cognitive processes remain to be examined.

The aim of the present study was to investigate whether task-irrelevant beliefs about the observed scene can modulate the sensory gain control. In our paradigm, attention was guided to a location by the gaze direction of a centrally presented face, and we manipulated beliefs regarding whether the face's gaze behavior resulted from the operations of a mind or of a machine. Crucially, these beliefs were entirely irrelevant to the task, and were manipulated either by cue identity (Experiment 1: presenting a human or a robot face) or solely by instruction, with cue identity remaining identical across conditions (Experiment 2: presenting only a robot face but informing participants that its gaze behavior was either human-controlled or pre-programmed). We reasoned that attentional control over sensory gain would be enhanced when the gaze behavior was believed to result from the operations of a mind, rather than a machine, as attending to locations gazed-at by an intentional agent is adaptive from the social and evolutionary perspective. Our reasoning followed Tomasello's distinction between two types of intention communicated through gaze: *referential* and *social* [21]. The first concerns the *object* of attention, the second *why* attention is directed to this object. The idea is that gaze behavior is usually only informative when it originates from a mind, because mental states not only cause gaze behavior, but also give meaning to it (the “why”). Consequently, if observers believe that an agent with a mind is directing gaze to a location, they may expect something relevant for communication at that location, and thus allocate their attention there. By contrast, if observers believe that a machine is directing “eyes” to a location, they may not allocate their attention there because the machine's gaze behavior is not attributable to the operations of a mind and thus lacks communicative content. Previous findings showed that gaze-guided attentional orienting can be modulated by attribution of particular mental states to the observed agent [22], [23]. Moreover, Wiese, Wykowska, and colleagues [24] showed that the general likelihood of attributing mind towards an observed agent influences gaze cueing effects. However, the present study is the first one designed to examine how neural mechanisms underlying gaze-related attentional orienting are modulated by higher-order (task-irrelevant) cognitive processes, such as beliefs about the observed scene. Importantly, by focusing on the sensory gain control, which is a mechanism of early selection, we aimed at showing flexibility in the early stages of perceptual processing.

## General Methods

### Ethics Statement

The experiments were conducted at the LMU Munich Department of Psychology (Laboratory of Experimental Psychology), where all experimental procedures that involve data collection from healthy adult participants and that do not involve invasive or potentially dangerous methods have been approved by the Department's ethics committee in accordance with the Code of Ethics of the World Medical Association (Declaration of Helsinki). Data were stored and analyzed anonymously. Participants gave their informed written consent and were either paid or received course credit for participating.

### Stimuli and apparatus

Stimuli were presented on a 17-inch computer screen with a 100-Hz refresh rate, placed at a distance of 80 cm from the observer. In the human-face condition of Experiment 1, a digitized photo, 5.7°×5.7° of visual angle in size, of the face of the same female individual, chosen from the Karolinska Directed Emotional Faces (KDEF, [25]) database (face F 07), was used, see [24] for the illustration of the female face stimulus. In the robot-face condition of Experiments 1 and 2, a photo of an anthropomorphic robot (EDDIE, LSR, TU München), of the same size as the human face, was presented.

**Figure 1:**
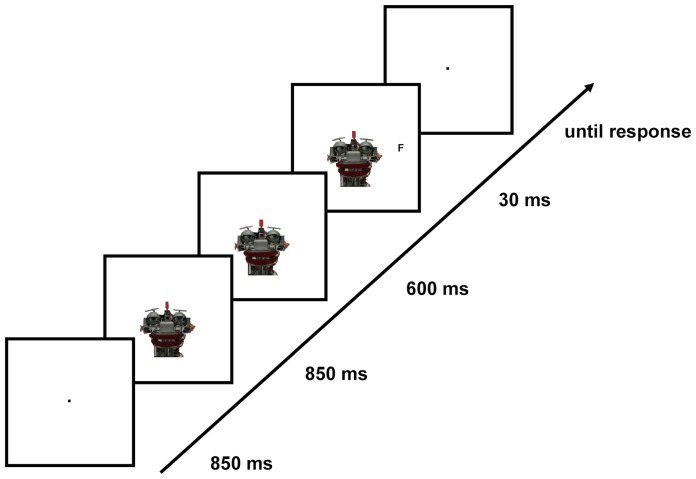
An example trial sequence. Participants first fixated on a fixation dot for 850(Experiment 1) or always a robot face (Experiment 2) gazing straight-ahead was presented for another 850 ms. Next, the gaze direction changed to either the left or the right for another 600 ms, which was then followed by target presentation (30 ms) either at the gazed-at location (valid-cue trial) or the opposite location (invalid-cue trials). Participants were then asked to respond to target identity, with a blank screen presented until the response. On catch trials, the display with a face gazing to the left/right was presented for another 30 ms. The stimuli are depicted as presented on the computer screen, with black outline squares representing the screen. The face stimuli were always presented with eyes at the level of the vertical midline of the screen, and at the same level as the target stimulus.

Both human and robot faces were presented frontally without changes in head orientation. To produce gaze direction cues, irises and pupils within the eyes were shifted (using Photoshop_TM_) left- or rightwards to deviate by 0.2° from straight-ahead gaze, in both the human and the robot condition. Stimuli were presented centrally on a white background, with eyes positioned on the central horizontal axis of the screen. The midpoints of the human and robot faces were positioned 0.2° and, respectively, 1.1° below the central horizontal axis; this slight difference in positioning with respect to the y-axis ensured that the peripheral target letters were always presented at the same level as the eyes of the human or robot face on the central horizontal axis. The target stimulus was a black capital letter (F or T), 0.2°×0.2° in size, which was presented on the central horizontal axis at an eccentricity of 5.7° with respect to the screen center (Figure 1). Target positions (left or right) were determined pseudo-randomly such that targets appeared with equal frequency at either of the two positions.

Gaze direction was not predictive of the target position, that is: in Experiment 1, on target-present trials (80% in total), gaze was directed either to the side on which the target appeared (valid trials, 33% of target-present trials) or to the other side (invalid trials, 33% of target-present trials), or it remained gazing straight-ahead, with targets equally likely appearing on either side (neutral trials, 33% of target-present trials). The neutral condition was introduced in order to examine for possible differential effects related to physical dissimilarities between the human and robot conditions. In Experiment 2, neutral trials were not included in the design. That is, the face could gaze to only the left or the right (50% trials with each direction, in target-present trials), with the target presented either on the right or on the left side of the screen. In both experiments, 20% of all trials were catch trials (no target presented). These target-absent trials were introduced to permit subtraction of the EEG signal on target-absent trials from that on target-present trials, so as to eliminate ERP potentials elicited by the cue, which overlapped with potentials related to the target.

### Experimental design

A trial started with a fixation point (2 pixels) presented for 850 ms. Subsequently, a face with gaze directed straight-ahead (towards the observer) appeared on the screen for 850 ms, while the fixation dot remained visible (in-between the face's eyes). The straight-ahead gazing face was followed by a gaze shift (cue) to the left or the right (valid and invalid trials), or the gaze remained straight ahead (neutral trials) for another 600 ms. Next, the target letter was presented on either the left or the right side of the screen, at a fixed stimulus onset asynchrony (SOA) of 600 ms relative to the onset of the gaze cue. Following this event, the face and target remained on the screen for another 30 ms only, in order to minimize eye movements in the critical time window. Participants were then asked to respond as quickly and accurately as possible to the identity of the target letter (F or T) using the ‘o’ or, respectively, the ‘i’ key on a standard keyboard (all other letters were removed and the o/i letters were covered with green/blue stickers), with response assignment (o = F/i = T vs. o = T/i = F) counterbalanced across participants. The keys were to be pressed with the index finger of the left and the right hand, respectively. The display was blank for the duration of response. Upon response, another trial started with the presentation of the fixation dot in the screen center. On target-absent trials, no response was required and the trial continued (blank screen) for another 800 ms. Participants were informed that the gaze (shift) direction of the cue provider was not predictive with respect to the actual target position, in either the human or robot face conditions of Experiment 1 or, respectively, the human-controlled or pre-programmed robot conditions of Experiment 2. For an illustration of the trial sequence, see Figure 1.

### EEG recording

EEG was recorded with Ag-AgCl electrodes from 64 electrodes of an active-electrode system (ActiCap, Brain Products, GmbH, Munich, Germany), at a sampling rate of 500 Hz. Horizontal and vertical EOG were recorded bipolar from the outer canthi of the eyes and from above and below the observer's left eye, respectively. All electrodes were referenced to Cz and re-referenced offline to the average of all electrodes. Electrode impedances were kept below 5 kΩ, and the EEG activity was amplified with a band-pass filter of 0.1 to 250 Hz using BrainAmp amplifiers (Brain Products, GmbH, Munich).

## Experiment 1

### Participants

Sixteen volunteers took part in the Experiment 1 (5 women; mean age: 24 years; age range: 20 to 30 years; all right-handed; and all with normal or corrected-to-normal vision; none of the observers had taken part in an experiment with such a paradigm before); they received an honorarium for their participation. The experiment was conducted with the full understanding and written consent of each participant. Experimental procedures were in accordance with the Code of Ethics of the World Medical Association (Declaration of Helsinki). Data of two participants had to be discarded due to technical problems during recording of the EEG data.

### Procedure

Participants were seated in a dimly lit chamber with a keyboard under their hands. Experiment 1 consisted of 900 trials and all conditions were randomly mixed within 10 blocks of 90 trials each. No specific instruction was given to participants regarding the type of cue (human vs. robot).

### Data analysis

We hypothesized that the directional gaze shift would guide attention to the gazed-at location. Hence, we expected validity effects (superior performance, and enhanced amplitudes of the P1-N1 ERP complex, for valid- vs. invalid-cue trials). Moreover, we expected the validity effects to be modulated by cue type – the rationale being that gaze following makes more sense if the gaze potentially conveys communicative content, relative to when it only reflects mechanistic behavior. In sum, the main factors of interest for all our analyses were: cue validity (valid vs. invalid) and cue type (human vs. robot). The analyses focused on valid and invalid trials, as neutral trials did not constitute a proper baseline – owing to the fact that in gaze cueing paradigms with naturalistic stimuli, neutral, straight-ahead gaze towards the observer is special in that it may induce an arousal effect and/or exert a holding effect on attention, making it difficult to disengage attention (from the central, straight-ahead gazing face) and shift it to the peripheral target [24], [26], [27]. Neutral trials were only analysed with respect to main effect of cue type, in order to examine for differential effects related to physical dissimilarities of the cue stimuli.

#### EEG data

The data were averaged over a 700-ms epoch including a 200-ms pre-stimulus baseline, with epochs time-locked to target onset. Trials with eye movements and blinks on any recording channel (indicated by any absolute voltage difference in a segment exceeding 80 μV or voltage steps between two sampling points exceeding 50 μV) were excluded from analyses. Additionally, channels with other artefacts were separately excluded if amplitude exceeded ±80 μV or any voltage was lower than 0.10 μV for a 100-ms interval. Only trials with correct responses were analyzed. No off-line filters were applied for analyses (30-Hz filters with 24 dB/Oct slope were applied to grand averages only for purposes of illustration). One participant was excluded from analyses due to extensive eye blinks. For each of the conditions of interest, there were 120 repetitions, with, on average, 92 repetitions remaining after rejection of eye movement artefacts (human valid: 91 trials; human invalid: 90 trials; human neutral: 93 trials; robot valid: 91 trials; robot invalid: 92 trials; robot neutral: 94 trials). For target-absent (catch) trials, there were 90 trials for the human-face and 90 for the robot-face condition, with 67 remaining on average in each condition after eye movement artifact rejection. Analyses were conducted on correct target-present trials with ERPs time-locked to target onset. The two types of target (F and T) as well as the side of presentation (left and right) were averaged together. Target-absent (catch) trials were subtracted from target-present trials, to eliminate overlapping potentials related to gaze cue onset and, thus, extract the potentials related to the targets. The subtraction was conducted on epoched data, separately for each type of cue (human vs. robot), each gaze direction (left vs. right), time-locked to target onset. The analyses focused on the comparison between valid and invalid trials. The EEG signal was averaged for the two validity conditions (valid vs. invalid) and the two types of cue (human vs. robot). We defined two regions of interest: left and right posterior-occipital regions, by averaging activity at PO7 and O1 electrodes for the left region and PO8 and O2 electrodes for the right region. Mean amplitudes in the typical time window of the P1 (100–140 ms) and N1 (150–190 ms) were subjected to ANOVAs with the factors *electrode site* (left vs. right), *cue type* (human vs. robot), and *cue validity* (valid vs. invalid). The P1 component time window (120 ms±20 ms) was selected based on grand average peak amplitude (120 ms) in the 100–150-ms time window in the human valid condition, where the P1 was most pronounced and where this component is typically observed [28]. The N1 component time window (170 ms±20 ms) was selected based on the latency of the grand average peak amplitude (170 ms) in the 140–200 ms time window in the robot valid condition, where the N1 component was most pronounced and where this component is typically observed [28]. Where appropriate, statistics were corrected according to Greenhouse-Geisser for potential non-sphericity. Planned comparisons were conducted for the valid vs. invalid conditions in the human and robot face conditions separately with one-tailed t-tests, due to directed a priori hypothesis regarding the validity effect: validly cued targets should elicit enhanced amplitudes for the P1/N1 time windows relative to invalidly cued targets [10], [18]–[20].

#### Behavioral data

Prior to the reaction time (RT) analysis, trials with response errors or RTs faster than 150 ms and longer 1200 ms were excluded. Median RTs and mean error rates were computed for each participant. The statistical analyses focused on the comparison between valid and invalid trials. Individual median RTs and mean error rates were submitted to a 2×2 analysis of variance (ANOVA) with the factors *cue type* (human vs. robot) and *cue validity* (valid, invalid). Planned comparisons were conducted for the valid vs. invalid conditions in the human and robot face conditions separately with one-tailed t-tests, due to directed a priori hypothesis regarding the validity effect: validly cued targets should elicit better performance than invalidly cued targets [5]–[9], [24]. The participant who was excluded from EEG analysis due to extensive eye blinks was also excluded from the behavioral analyses, as the frequent blinking could, in general, have affected visual processing of the (briefly presented) target stimuli.

### Results

#### ERP data

**Figure 2:**
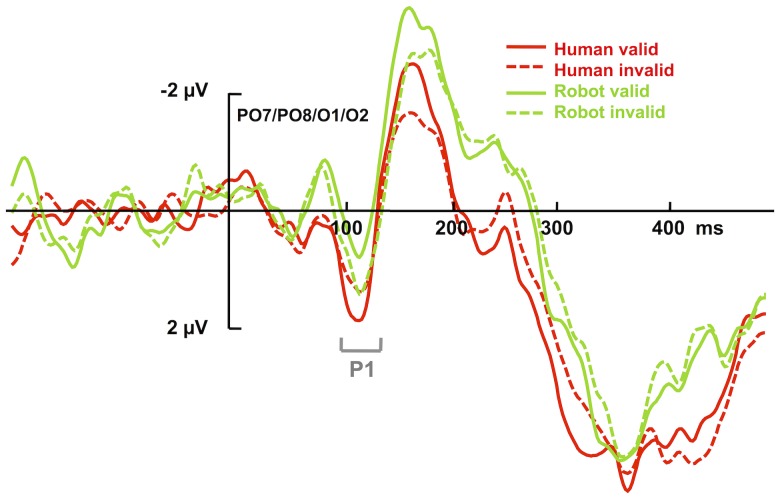
Grand average ERP waveforms time-locked to target onset and voltage distributions in Experiment 1. The depicted waveforms (left) represent ERPs for the pool of O1/O2/PO7/PO8 electrodes, as a function of cue validity (solid lines: valid trials; dashed lines: invalid trials) and type of cue provider (red: human faces, green: robot faces), in Experiment 1. The two types of targets (F and T) as well as left/right sides of visual field were averaged together. The displayed ERPs are the subtracted waveforms (target present–target absent) and filtered with a 30-Hz high cut-off filter (Butterworth zero phase, 24 dB/Oct) for illustration purposes.

**Figure 3:**
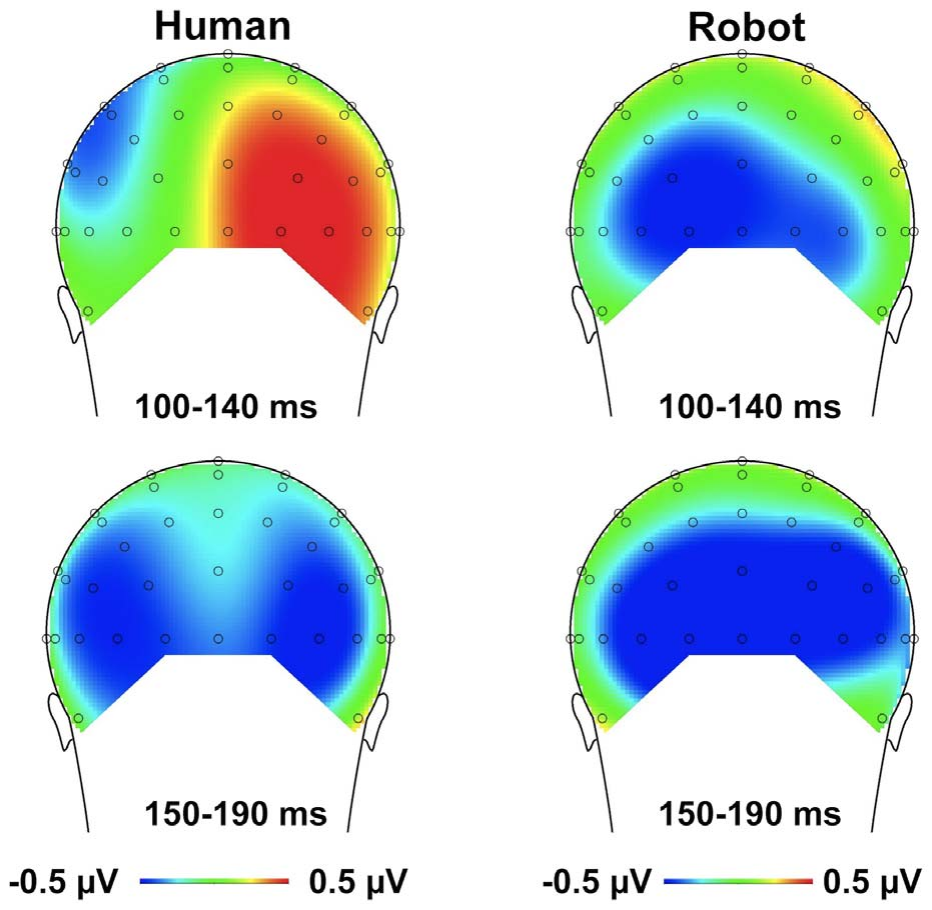
Topographical maps of voltage distribution (mean amplitude) for the *difference* between the valid and invalid conditions for the human face (left) and the robot face (right). The time interval of the P1 component (100–140 ms) is presented in the upper panel; the time interval of the N1 component (150–190 ms) is presented in the lower panel. Voltage distribution maps are presented from posterior view.

The 2×2×2 ANOVA of the mean amplitudes in the P1 time window (100–140 ms), with the factors *cue validity* (valid vs. invalid), *cue type* (human vs. robot), and *electrode site* (left vs. right), revealed the cue type x cue validity interaction to be significant, *F* (1, 12) = 7.922, *p* = .016, *η*_p_^2^ = .398, and uninfluenced by electrode site (three-way interaction with electrode site: *p* = .31). The main effect of cue type was not significant, *F* (1, 12) = 1.04, *p* = .327. Note though that planned comparisons conducted separately for the human- and robot-face conditions yielded only marginally significant validity effects: (i) for the human-face condition, the P1 amplitude was more positive for valid than for invalid trials (*M_valid_* = 1.27 μV, *SEM* = .44 vs. *M_invalid_* = .93 μV, *SEM* = .43; *t* (12) = 1.52, *p* = .075, one-tailed; see Figure 2); (ii) for the robot face condition, the validity effect tended to be reversed, with a slightly less positive P1 amplitude for valid than for invalid trials (*M_valid_* = .31 μV, *SEM* = .45 vs. *M_invalid_* = .78 μV, *SEM* = .36; t (12) = 1.77, p = .05, one-tailed; see Figure 2). However, a more clear-cut picture emerged when the factor electrode site was included in the (separate) analyses of the cue validity effects. In the human-face condition, the validity effect interacted with electrode site, *F* (1, 12) = 13.524, *p* = .003, *η*_p_^2^ = .53: there was a significant validity effect for the right posterior-occipital site, *F* (1, 12) = 8.584, *p* = .013, *η*_p_^2^ = .417, but not for the left site, *p* = .93 (see Figure 3). The robot-face condition, by contrast, did not yield any significant main effects or interactions of interest (validity effect: *p* = .103; validity × electrode site interaction: *p* = .977). Note that there was no indication that the effects of interest were lateralized in relation to side of target presentation: an ANOVA that included the factor target side (left vs. right) in addition to electrode site (left vs. right), validity (valid vs. invalid), and cue type (human vs. robot) yielded no evidence of a significant four-way interaction, *p* = .997. Note further that visual inspection of the grand-averaged ERP waveforms suggested a differential effect in a time window preceding that of the P1 component, on the negative deflection of the waveform; see Figure 2. However, statistical analysis on this time window (60–100 ms) failed to yield any significant effects; in particular, the interaction of cue and validity was non-significant, *F* = .009, *p* = .926 (cue type, *F* = 1.609, *p* = .229, and validity, *F* = .031, *p* = .862).

An analogous analysis for the later time window (150–190 ms) of the N1 ERP component revealed a main effect of cue validity, *F* (1, 12) = 8.059, *p* = .015, *η*_p_^2^ = .402, with valid trials eliciting a more negative mean amplitude (*M* = −2.33 μV, *SEM* = .5) compared to invalid trials (*M* = −1.7 μV, *SEM* = .58). This effect was not influenced by the type of cue, *p* = .79, or by electrode site, *p* = .257; see Figure 2.

#### Behavior

The 2×2 ANOVA with the factors of *cue type* (human vs. robot face) and *validity* (valid vs. invalid) on error rates revealed the interaction to be significant, *F* (1, 12) = 5.902, *p* = .032, *η*_p_^2^ = .33, with the validity effect being more pronounced for human than for robot faces (ΔER = 2% vs. ΔER = −0.4%). Planned comparisons showed that for the human-face condition, error rates were significantly lower for valid than for invalid trials (*M_valid_* = 3.8%, *SEM* = .8 vs. *M_inalid_* = 5.7%, *SEM* = .9; *t* (12) = 2.44, *p* = .015, one-tailed); by contrast, there was no difference between these two types of trial in the robot-face condition (*M_valid_* = 5.2%; *M_invalid_* = 4.8%; p = .353, one-tailed).

An analogous analysis on the median RTs revealed no significant main effects or interactions. Numerically, RTs were overall slightly faster for valid than for invalid trials (*M_valid_* = 404 ms, *SEM* = 11 vs. *M_invalid_* = 410 ms, *SEM* = 10), though the main effect of validity was not significant: *F* (1, 12) = 3.46, *p* = .088, *η*_p_^2^ = .224. Also, the difference was numerically larger for human faces (RT_valid_ = 405 ms; RT_invalid_ = 411 ms) than for robot faces (RT_valid_ = 404 ms; RT_invalid_ = 408 ms), though not reliable statistically (cue type × cue validity interaction: *F* (1, 12) = .421, *p* = .529).

When both behavioral measures (RTs and accuracy) were combined into a single dependent variable, namely: “inverse efficiency scores (IES)” [29], [30], by dividing individual median RTs by individual accuracy scores (percentages of correct responses), a 2×2 ANOVA with the factors *cue type* (human vs. robot face) and *validity* (valid vs. invalid) revealed the interaction to be marginally significant, *F* (1, 12) = 4.536, *p* = .055, *η*_p_^2^ = .274, with the validity effect being more pronounced for human faces (ΔRT = 15 ms) than for robot faces (ΔRT = 3 ms). Planned comparisons showed that for the human-face condition, the cue validity effect was significant, *t* (12) = 2.739, *p* = .009, one-tailed; by contrast, there was no significant effect for the robot-face condition *t* (12) = .507, *p* = .311, one-tailed.

In order to examine whether physical dissimilarity between human and robot gaze cues as such (or the slightly different positioning of the human vs. robot face stimuli on the vertical axis of the computer screen) has an influence on the amplitude of the early sensory P1 component, we compared the neutral-trial P1 mean amplitude between the human- and robot-face conditions. A 2×2 ANOVA with the factors *electrode site* (left vs. right) and *cue type* (robot vs. human) revealed no main effect of cue type, *F* (1, 12) = .093, *p* = .766, and no interaction with electrode site, *F* (1, 12) = .229, *p* = .641. The behavioral data, too, showed no indication of differential processing on neutral trials between robot and human faces: *t* (12) = .422, *p* = .68 for the error rates, and *t* (12) = .628, *p* = .542 (two-tailed) for the median RTs; see Table 1 for the mean RTs, error rates, and P1 amplitudes in the neutral condition.

**Table 1:** Mean RTs, error rates and mean amplitude of the P1 component (100–140 ms time window) for the neutral cue condition (gaze straight-ahead) as a function of type of cue provider (human vs. robot).

	Median RTs	Mean error rates	Mean P1 amplitude
Human	425 ms (12)	4.15% (.67)	1.223 μV (.68)
Robot	427 ms (10)	3.85% (.67)	1.379 μV (.53)

Standard errors of the mean are provided in brackets.

## Experiment 2

Experiment 2 was designed to eliminate the possibility that physical differences between the two types of cue providers (or their slightly different positions on the computer screen) were responsible for the pattern of effects observed in Experiment 1, rather than differences in mind-attribution. This alternative explanation is unlikely, given that: (i) a comparison of the neutral trials between the human and the robot condition did not reveal any significant P1 effects; (ii) valid trials were compared with invalid trials within each of the two cue type conditions (robot, human), rather than across conditions; and (iii) no main effect of cue type was observed in either in the P1 or the N1 time window, indicating that the amplitudes of those components were not influenced by the type of stimulus as such. Nevertheless, it remains critical to positively demonstrate that the modulation of sensory gain control is attributable solely to the higher-order belief that the gaze behavior was the result of the operations of a mind, rather than of a machine; in other words, to experimentally isolate this top-down modulation in order to rule out alternative explanations of the P1 effect. To achieve this, in Experiment 2, we used only one, physically identical cue provider across all conditions while manipulating participants' beliefs via instruction. Specifically, participants were presented with the same robot face (gazing to the left or the right; see Figure 1) across all conditions. Crucially, in one experimental session, they were told that the robot's gaze behavior was pre-programmed (Instruction 1), and in the other session, they were told that the eyes of the robot were controlled by a human (Instruction 2).

### Participants

Twenty-eight volunteers took part in the Experiment (19 women; mean age: 24.4 years; age range 19 to 34 years; 7 left-handed; all with normal or corrected-to-normal vision; none of the observers had taken part in any other experiment with such a paradigm). Participants received an honorarium for their participation. The experiments were conducted with written consent of each participant. Experimental procedures were in accordance with the Code of Ethics of the World Medical Association (Declaration of Helsinki).

### Procedure

Participants were seated in a dimly lit chamber with a keyboard under their hands. Trial sequence was identical to that of Experiment 1, except that only one type of gazer was presented (robot face) and there were no neutral-cue trials (i.e., all gaze cues were either valid or invalid on target-present trials). There were altogether 960 trials, split into 2 sessions with two different instructions (on the same day, with a 15–30 min break in between). Each of the participants received both instructions (Instruction 1: human-controlled, Instruction 2: pre-programmed), with order counterbalanced across participants. Instructions were provided to participants in German, in written form. Instruction 1 stated: “In this experiment, a picture of a robot will be displayed, whose eye movements are in fact performed by a human. The human's eye movements are directly transferred in real-time to the robot face through a computer. This way, the robot's eyes can be controlled by a human”. Instruction 2 read: “In this experiment, a picture of a robot will be displayed, whose eye movements have been pre-programmed, so that they move according to a pre-defined template”. Participants who received Instruction 1 in Session 1 received Instruction 2 in Session 2, with the additional information: “The only difference from the first session of this experiment is that in the present session, the eyes of the robot will be controlled by a computer program, and not by a human”; and participants who received Instruction 2 in Session 1 read Instruction 1 in Session 2, with the additional information: “The only difference from the first session of this experiment is that in the present session, the eyes of the robot will be controlled by a human, and not by a computer program”. The instructions also specified the task (discrimination of the letters), key assignment, number of blocks with estimated time; and provided pictures of the face stimulus. They also stated that gaze direction of the robot face would not be predictive of the target location in either the human-controlled or the pre-programmed conditions.

### Data analysis

We expected the validity effects (superior performance, and enhanced amplitudes of the P1-N1 ERP complex, for valid- vs. invalid-cue trials) to be modulated by instruction; thus, the main factors of interest for all our analyses were: cue validity (valid vs. invalid) and instruction (human-controlled vs. pre-programmed).

#### EEG data

The data were averaged over a 500-ms epoch (+200-ms pre-stimulus baseline), time-locked to target onset. Trials with eye movements and blinks on any recording channel were excluded from analyses (absolute voltage difference in a segment exceeding 80 μV or voltage steps between two sampling points exceeding 50 μV on VEOG or HEOG). Additionally, channels with other artefacts were excluded if amplitude exceeded ±80 μV or any voltage was lower than 0.10 μV for a 100-ms interval. No off-line filters were applied for analyses (30-Hz filters with 24 dB/Oct slope were applied to grand averages only for purposes of illustration). Three participants were excluded from analyses due to extensive eye blinks, and one due to abnormal alpha activity. None of the remaining participants exhibited eye movements deviating more than .2° from central fixation during the cue-target interval (average differential activity, leftward-gaze trials subtracted from rightward-gaze trials, on either of the HEOG channels, F9 or F10, did not exceed 3.3 μV during presentation of the face with directed gaze; for the procedure see [31]). One further participant was excluded from analysis due to residual eye movement activity after artefact rejection in the target-locked interval (differential activity on the HEOG channels (right target vs. left target) exceeded 3.3 μV, but did not exceed 5 μV =  eye movements deviating from fixation <.3° [32]). For each of the conditions of interest, there were 192 repetitions, with 171 repetitions remaining on average after eye movement rejection (human-controlled valid: 173 trials; human-controlled invalid: 180 trials; pre-programmed valid: 170 trials; pre-programmed invalid: 161 trials).

Analyses were conducted on correct target-present trials with ERPs time-locked to target onset. The two types of target (F and T) as well as the side of presentation (left and right) were averaged together. Target-absent (catch) trials were subtracted from target-present trials to eliminate overlapping potentials related to gaze cue onset and, thus, to isolate the potentials related to the targets. The subtraction was conducted on epoched data, separately for each type of instruction and each gaze direction, time-locked to target onset. The EEG signal was averaged for the two validity conditions and the two types of instruction. Mean amplitudes in the time window of the P1 component (100–140 ms, i.e., ±20 ms from the latency of the grand average peak amplitude in the 100–150-ms time window in the human valid condition, in which P1 was most pronounced; regarded as the typical P1 time window, in line with [28]), as well as in the subsequent window of the N1 component (170–210 ms in Experiment 2; i.e., ±20 ms from the latency of the grand average peak amplitude in the 140–200-ms time window in the robot valid condition, in which the N1 was most pronounced) for the lateral posterior-occipital electrode sites (left: O1/PO7 vs. right: O2/PO8) were subjected to ANOVAs with the factors *electrode site* (left vs. right), *instruction* (human-controlled vs. pre-programmed), and *cue validity* (valid vs. invalid). Where appropriate, statistics were corrected according to Greenhouse-Geisser for potential non-sphericity. Planned comparisons of valid- vs. invalid-cue trials were performed separately for the human-controlled and pre-programmed conditions using one-tailed t-tests, given directed a-priori hypotheses regarding the validity effects: validly cued targets should elicit enhanced amplitudes in the P1/N1 time windows relative to invalidly cued targets [10], [18]–[20]. The average differential activity (target left – target right) on HEOG channels was examined for the time windows of interest (100–140 ms and, respectively, 170–210 ms post target onset) as a function of cue validity and instruction. Neither the main effect of validity nor the interaction of validity and instruction were significant, for both windows of interest (both Fs<1.4; ps>.25).

#### Behavioral data

Prior to the reaction time (RT) analysis, trials with response errors or RTs faster than 150 ms (regarded as anticipations) and longer 1200 ms (regarded as exceptionally long responses) were excluded. Median RTs and mean error rates were computed for each participant. Individual median RTs and mean error rates were submitted to a 2×2 ANOVA with the factors *instruction* (human-controlled, pre-programmed) and *cue validity* (valid, invalid). Planned comparisons of valid- vs. invalid-cue trials were performed separately for the human-controlled and pre-programmed conditions using one-tailed t-tests, given directed a-priori hypotheses regarding the validity effects: validly cued targets should elicit better performance relative to invalidly cued targets [5]–[9], [24]. The three participants who exhibited excessive blink or eye movement artifacts in the EEG data, which might have influenced visual processing of the stimuli, were also not included in the behavioral analyses, too.

### Results

#### ERP data

**Figure 4:**
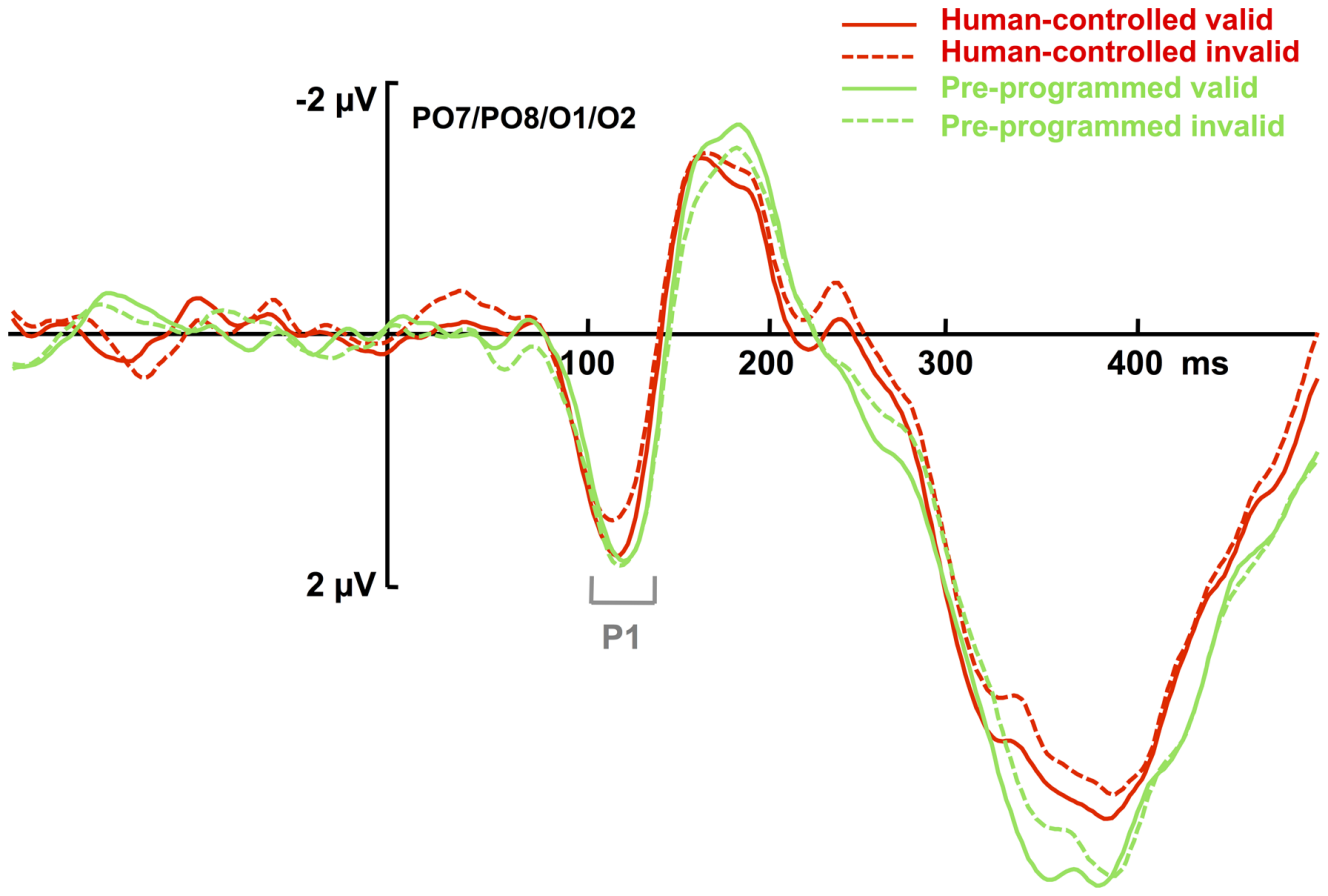
Grand average ERP waveforms time-locked to target onset in Experiment 2. The depicted waveforms represent ERPs for the pool of O1/O2/PO7/PO8 electrodes, as a function of cue validity (solid lines: valid trials; dashed lines: invalid trials) and instruction (black: human-controlled, gray: pre-programmed). The two types of targets (F and T) as well as left/right sides of visual field were averaged together. The displayed ERPs are the subtracted waveforms (target present-target absent) and filtered with a 30-Hz high-cutoff filter (Butterworth zero phase, 24 dB/Oct) for illustration purposes.

The 2×2×2 ANOVA of the mean amplitudes in the P1 time window (100–140 ms), with the factors *cue validity* (valid, invalid), *instruction* (human-controlled, pre-programmed), and *electrode site* (left vs. right), revealed the validity x instruction interaction to be significant, *F* (1, 22) = 8.426, *p* = .008, *η*_p_^2^ = .277, with a significantly more positive P1 amplitude for valid than for invalid trials in the *human-controlled* condition (*M_valid_* = 1.48 μV, *SEM* = .3 vs. *M_invalid_* = 1.27 μV, *SEM* = .3), *t* (22) = 1.78, *p* = .044, one-tailed, and a slightly (non-significantly) less positive amplitude for valid than for invalid trials in the *pre-programmed* condition (*M_valid_* = 1.41 μV, *SEM* = .4 vs. *M_inalid_* = 1.53 μV, *SEM* = .4), *t* (21) = 1.03, *p* = .15, one tailed (Figure 4).

**Figure 5:**
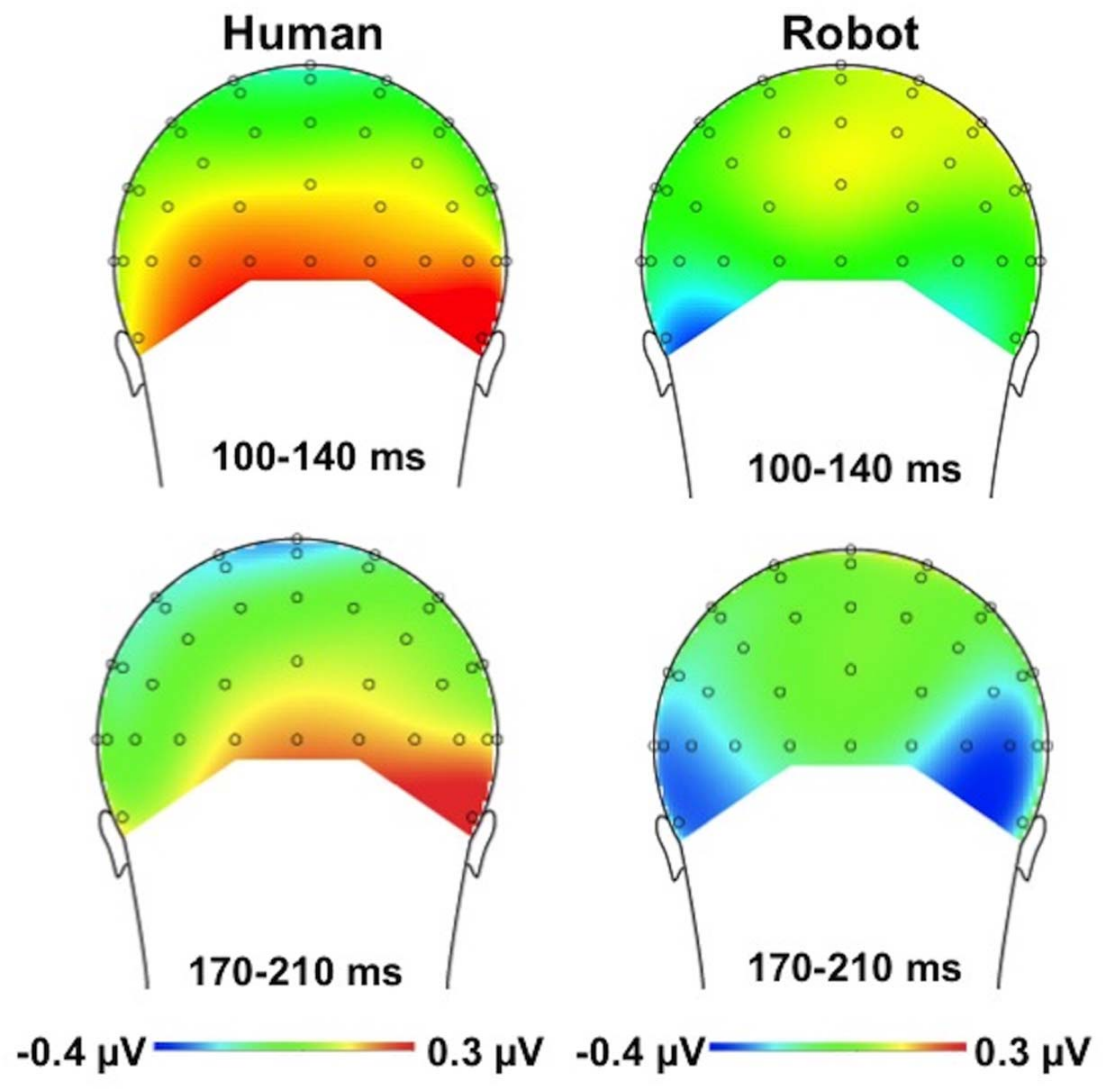
Topographical maps of voltage distribution (mean amplitude) for the *difference* between the valid and invalid conditions for the human-controlled condition (left) and the pre-programmed condition (right). The time interval of the P1 component (100–140 ms) is presented in the upper panel; the time interval of the N1 component (170–210 ms) is presented in the lower panel. Voltage distribution maps are presented from posterior view.

The interaction between cue validity and instruction was not influenced by electrode site (three-way interaction with electrode: *F*(1, 22) = .04, *p* = .844); see Figure 5 for the voltage distribution. There was no indication that the effects of interest were lateralized in relation to side of target presentation: an ANOVA that included the factor target side (left vs. right) in addition to electrode site (left vs. right), validity (valid vs. invalid), and instruction (human-controlled vs. pre-programmed) yielded no evidence of a significant four-way interaction, *p* = .684.

An analogous analysis on the mean amplitudes in the N1 time window (170–210 ms) revealed no main effect of validity, *F* (1, 22) = .153, *p* = .699, and no significant interaction of validity and instruction, *F* (1, 22) = 2.683, *p* = .116.

#### Behavior

A 2×2 ANOVA of the median RTs with the factors *instruction* (human-controlled, pre-programmed) and *cue validity* (valid, invalid) showed that the ERP effects were paralleled in the behavioral data: instruction type interacted with cue validity, *F* (1, 24) = 5.47, *p* = .028, *η*_p_^2^ = .186; with the validity effect being significant in the *human-controlled* condition, *t* (24) = 2.071, *p* = .025, one-tailed (*M_valid_* = 408 ms, *SEM* = 9 vs. *M_invalid_* = 411 ms, *SEM* = 10), but not in the *pre-programmed* condition, *t* (24) = .886, *p* = .192, one-tailed (*M_valid_* = 410 ms, *SEM* = 9 vs. *M_invalid_* = 409 ms, *SEM* = 10). Analogous analyses on the error rates and inverse efficiency scores revealed no significant effects or interactions, all *F*s<2, *p*s>.18 (error rates) and all *F*s<3.3, *p*s>.08 (inverse efficiency scores).

## General Discussion

The purpose of the present study was to investigate whether early sensory processes are penetrable by higher-order cognitive processes, such as beliefs about the observed scene. We examined for modulations of the attention-related sensory gain control mechanism with attention being guided by gaze. In our paradigm, attentional orienting was induced by gaze shifts, and beliefs about the observed gazer were manipulated either by the identity of the face (Experiment 1) or solely by instruction, with the gazer's identity remaining identical across conditions (Experiment 2). We hypothesized that attentional control over sensory processing (the sensory gain control) would be enhanced when participants believed that the observed gaze behavior was controlled by a mind, rather than by a machine.

Our data support this hypothesis: In two experiments, the target-locked P1 was more enhanced for the valid-cue, relative to invalid-cue, trials, but only when the gazer's behavior was believed to result from operations of a mind. This ERP effect was paralleled by the behavioral data: target-related performance was better on valid-cue, relative to invalid-cue, trials when participants believed the gazer had a mind and was not a machine, replicating previous behavioral results [24] in a within-participants design. The ERP and behavioral data are particularly intriguing because participants' beliefs about the gazer were completely irrelevant to the discrimination task they had to perform. Recall that participants in the present study were expressly informed that gaze (shift) direction was entirely non-predictive with respect to the target location, in all experimental conditions. Accordingly, the pattern of results obtained is unlikely attributable to participants having formed differential expectations about cue validity, dependent on whether they did or did not adopt the ‘Intentional Stance’ (see below) towards the gazer.

Interestingly, the N1 validity effect was not modulated by cue type in Experiment 1, and no validity or instruction effect on the N1 was observed in Experiment 2. The P1 and N1 components have previously been proposed to reflect different modes of control over sensory gain: the P1 has been argued to reflect a suppression mechanism for ignored locations, whereas the N1 indexes enhanced discriminative processing of stimuli at the attended locations [11], [18], [33]. Given this, the differential effects between the P1 and N1 suggest that when target stimuli are presented very briefly, higher-level cognitive processes influence only the earlier, suppression-related mechanism to increase the signal-to-noise ratio, but not the later, discriminative processes at the attended locations.

In sum, this is the first study to show that higher-order, task-irrelevant beliefs about the observed scene can influence early sensory processing by modulating stimulus-related neuronal activity, dependent on whether the stimulus location has been signaled by a meaningful social cue (gaze direction of an agent with a mind) or not (gaze direction of a machine).

### Theoretical considerations

The present findings can be interpreted along the idea that humans adopt various “stances” in order to predict and understand behavior of various systems with which they interact: the Physical, the Design, or the *Intentional Stance* [34]. Based on experience, humans know which stance works best for which system. For example, when explaining the workings of a machine, it is best to adopt the Design Stance (DS) and understand its behavior with reference to how it is designed to behave. In contrast, when explaining other humans' behavior, the most efficient strategy is to engage in *mentalizing* [35]: predicting and understanding behavior with reference to particular mental states (e.g., beliefs, desires, intentions).

However, we argue that before one can engage in mentalizing (i.e., refer to any particular mental state), one needs to fundamentally assume that the entity whose behavior one is explaining is actually capable of having mental states. That is, one needs to adopt the *Intentional Stance* (IS) towards the observed entity by assuming that the entity has a mind. Our findings show that attentional control over sensory processing (sensory gain control) is exerted depending on whether or not one adopts the IS towards an observed entity.

**Figure 6:**
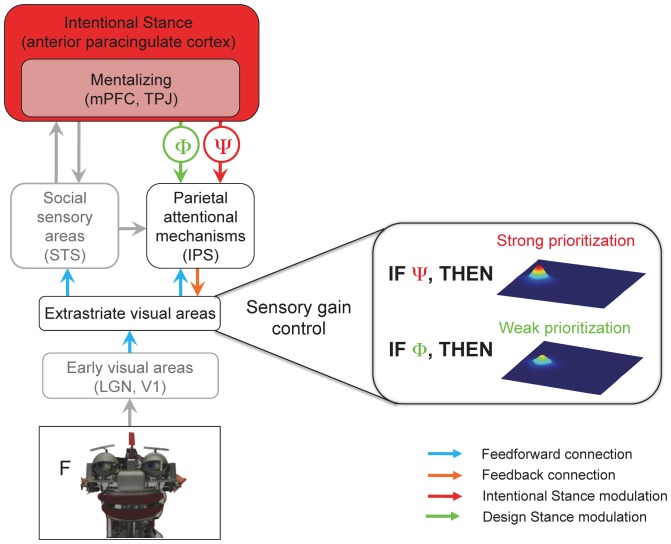
The Intentional Stance Model (ISM) of social attention. A visual stimulus (the robot face, bottom) is processed in the visual pathway from the lowest-level (early visual areas box) to higher-level areas (e.g., STS). The Attentional Network (IPS) is involved in orienting attention to the stimulus (the letter F) that is cued by the gaze. One of the core claims of ISM is that mentalizing is dependent on the Intentional Stance (IS), because it logically and functionally presupposes the adoption of the IS. Adopting the IS (or DS) occurs most probably in the anterior paracingulate cortex (36) and feeds back to the parietal attentional mechanisms, subsequently modulating the sensory gain control in the extrastriate visual areas (right). When observing an entity's gaze behavior while adopting the IS, this higher-order belief modulates the sensory gain control in the extrastriate areas, increasing the priority of an item cued by the gaze (represented by a higher peak of neural activity on the right; the other peak depicts an invalidly cued object). This additional prioritization does not occur when the brain adopts the DS. Thus, beliefs about the mind of others influence one's own mind. LGN =  lateral geniculate nucleus, V1 =  primary visual cortex, STS =  superior temporal sulcus, IPS =  intraparietal sulcus, mPFC =  medial prefrontal cortex, TPJ =  temporo-parietal junction. Processes of social cognition and perception that are the focus of this paper and are essential for the core claims of the ISM are highlighted in black and color, while gray boxes represent other processes of social perception/cognition that are not in the focus of this paper.

To account for these findings, we propose the *Intentional Stance Model* (ISM) of social attention (Figure 6). According to the ISM, when the brain adopts the IS towards *A*, *A* is represented as an agent with a mind. This representation allows for interpreting the behavior of *A* with reference to particular mental states (i.e., to mentalize). Importantly, the same behavior can be interpreted without reference to mental states, if one assumes that *A* is a mechanistic device and adopts the DS instead. For example, one can explain *A* gazing at an apple either with reference to mental states (*A wants* to eat the apple); or with reference to mechanistic states (*A*'s machinery shifts the camera lens around).

As a result, mechanisms of social attention will be deployed at various levels, dependent on whether IS is adopted or not; and, accordingly, prioritization of processing of an object falling within the focus of attention (the sensory gain control) will be engaged to a higher (IS adopted) or lower degree (DS adopted).

Previous research has shown that mentalizing influences perceptual processing [23]. Teufel and colleagues [23] proposed the so-called *perceptual mentalizing model* (PMM) to account for these mentalizing-dependent effects. According to PMM, when observers perform a gaze-cueing task, brain areas implicated in mentalizing: the medial prefrontal cortex (mPFC) and temporo-parietal junction (TPJ), generate signals which modulate neural activity in social perception areas, such as the superior temporal sulcus (STS). The STS in turn interacts with the parietal attention mechanisms of the intraparietal sulcus (IPS) in order to orient attention in the direction of the gaze by increasing the commitment of neural resources to the gazed-at location. One limitation of the PMM, however, is that it does not account for the impact of adopting the IS on sensory processing. As described above, mentalizing logically and functionally presupposes adopting the IS, because the brain must first assume that the observed entity is actually capable of having mental states before it can infer the mental states underlying particular behaviors.

The ISM overcomes this limitation by proposing a neurocognitive machinery by which adopting the IS exerts top-down influences on social attention, namely: feedback of IS predictions to lower levels of the processing hierarchy, modulating the *sensory gain control*. Based on the present findings, these modulations reach as low as the extrastriate visual areas, where stimulus coding is influenced by the sensory gain mechanism some 100 ms after gaze cue onset. Whether the IS modulations can take effect even earlier (before 100 ms) and in even lower visual areas, such as V1, remains to be established in future research. This might well be the case, as previous studies have shown that top-down control mechanisms can affect perceptual processing in areas as low as V1, as early as 55–90 ms after cue onset [37]–[39].

The functional necessity of modulatory predictions in social perception is also suggested by recent findings implicating the dorsal and ventral medial PFC and ventral striatum in the functional neuroanatomy underlying joint attention [40], [41]. This is noteworthy as the dorsal medial PFC is involved not just in mentalizing [23] but also in adopting the Intentional Stance [36], whereas the ventral medial PFC and ventral striatum are involved in reward predictions and value-based choices [42]–[44]. Given the strong evolutionary grounds for why social interactions may be intrinsically rewarding and valuable [41], [45], [46], future research should focus on elaborating the link among reward, predicted value, and social cognition/perception.

Furthermore, it is worth considering the ISM in light of the “second-person approach” to mentalizing [46], which stresses emotional engagement and social interactions with others as the driving mechanisms for mentalizing. On this view, knowledge of other minds emerges by virtue of being embedded in and coupled with the world in a particular manner [46], where emotional engagement refers to the degree of responsiveness between agents, and social interaction to the “reciprocal relations with the perception of socially relevant information prompting (re-) actions, which are themselves processed and reacted to” ([46], p. 397). While it is clearly important to take into account that the manner of our coupling with the environment determines how we understand other minds, ISM emphasizes that a purely embodied or embedded approach to mentalizing should not downplay the causal centrality of internal representations. This is because the very concepts of “emotion”, “responsiveness”, and “social relevance” presuppose – both logically and functionally – that the brain has represented the observed entity it is responding to and interacting with as being actually capable of having mental states (i.e., adopted the IS). This does not, however, preclude bottom-up signals (e.g., the behavior of the interacting partner) from driving mentalizing, or from having an influence on adopting the Intentional Stance. In fact, Pfeiffer et al. [47] showed that attribution of humanness (and thereby presumably adoption of the IS) depended on the observed behavior of an avatar and on prior beliefs regarding particular mental states underlying the behavior. It remains to be examined to what extent adopting the IS is driven by the bottom-up, interactive aspects of social cognition versus a-priori beliefs and assumptions. Importantly, what ISM proposes is that mentalizing functionally depends upon a particular type of higher-order representations that we collectively refer to as the “Intentional Stance”.

To conclude, the present study showed that a general perceptual selection mechanism – sensory gain control – is governed not just by intrinsically visual factors, such as spatial or feature-based selection, but is sensitive to higher-order task-irrelevant beliefs about others. This implies that mechanisms of early perceptual selection exhibit a high degree of flexibility and penetrability to top-down control.
